# NANOMATERIALS: Examining Nanotech’s Clean Energy Promises

**DOI:** 10.1289/ehp.119-a17

**Published:** 2011-01

**Authors:** Rebecca Kessler

**Affiliations:** **Rebecca Kessler**, based in Providence, RI, writes about science and the environment for various publications. She is a member of the National Association of Science Writers and the Society of Environmental Journalists

Among the many touted benefits of nanotechnology, one of the most alluring is the possibility that it will help reduce reliance on fossil fuels. Researchers and industry analysts foresee lighter and more efficient vehicles and wind turbines, solar panels that capture more of the sun’s energy, smaller and longer-lasting batteries, better insulation, and smarter lighting, to name a few nanotechnology prospects, some already on the market. But a new report from the conservation group Friends of the Earth (FOE) criticizes the vision of a clean-energy revolution brought about by engineered nanomaterials as so much greenwash and claims the young technology’s carbon, environmental, and human-health footprints are likely to eclipse any energy savings.^1^

Engineered nanomaterials are a relatively new class of manufactured materials with at least one dimension between 1 and 100 nm. The larger ones are about one-eightieth the size of a red blood cell. At such small scales, the ratio of surface area to volume is huge, giving the material novel properties. Nanomaterials in an array of shapes and chemistries are being applied to medicine, consumer products, environmental remediation, the energy industry, and more.

The FOE report focuses in part on the enormous amounts of energy needed to produce many nanomaterials. For instance, one life-cycle analysis calculated that carbon nanotubes, which are widely used to strengthen and lighten manufactured goods, require 2–100 times more energy to produce than aluminum, a notorious energy hog.^2^ But some critics of the report question whether the energy it takes to produce nanomaterials torpedoes their overall benefit. In a statement, Jay West, senior director of the Nanotechnology Panel at the American Chemistry Council, said, “[w]hile some nanomaterials may be energy-intensive to produce, such energy expenditures may be more than offset by the energy savings they make possible.” (Requests for comment on the report were declined by the U.S. Department of Energy.)

The FOE report also challenges whether nanotechnology will be able to deliver energy savings promised in a long list of applications quickly enough to make a difference. For instance, it cites several studies showing solar panels made with nanomaterials trail conventional silicon panels in efficiency and durability, and says there’s not a moment to spare waiting for nanotechnology to catch up. “With climate change we don’t really have that much time to ameliorate the situation,” says Ian Illuminato, one of the report’s authors. Moreover, the FOE report warns that petrochemical companies are investing heavily in nanotechnology in the hope it could double the amount of oil that can be extracted from known oil and gas reserves. It also points out that the manufacturing process for many nanomaterials relies on high inputs of water and solvents and generates hazardous by-products and a great deal of waste.

Yet David Rejeski, director of the Project on Emerging Nanotechnologies at the Woodrow Wilson International Center for Scholars, says, “Compared with the development times of other technologies, nano is not particularly slow and may even be faster. You could say that it has been moving at a pace that will make it unlikely to offer large-scale solutions to the climate challenge within the next five to ten years. But in ten to twenty years, nano will likely play a much larger role in terms of energy solutions.”

One thing everyone seems to agree on is that cost is a big reason for pursuing nanotechnology in the solar industry. Currently traditional silicon-based solar cells generate energy at a price of about $1.50–2.00 per watt.^3^ In order for solar to capture a substantial share of the energy market, however, the cost must go down significantly, and silicon-based panels have little hope of keeping up, says Ashok Sood, president and CEO of Magnolia Solar, a startup company developing nanostructure-based solar cells. He says his company’s business model relies on analyses and experimental data showing that such solar cells can meet or beat the efficiency of silicon-based cells, bringing the price per watt down to under $1.00. “Have they been demonstrated? Partially. Is the potential there? Yes. That’s what this is all about,” he says. “If I can do under one dollar a watt, I have a winner.”

There also is general agreement that much more information is needed about the potential human health effects of nanomaterials. The limited evidence to date gives some researchers pause. For example, several mouse studies have shown that carbon nanotubes injected into the abdominal cavity (a surrogate for human mesothelial exposure) or instilled into the trachea behave much like asbestos.^4^,^5^ Another study showed that nanoscale titanium dioxide administered subcutaneously to pregnant mice caused nerve damage in their offspring.^6^

FOE has been calling for a moratorium on the commercialization of products containing nanomaterials for the past five years, pending regulation to protect against potential threats to public health and the environment. “We need sound regulation, but unfortunately science and new technology always pose regulatory challenges that our agencies just aren’t prepared for. But at the same time, we’ve got thousands of products [already] on the market,” says Illuminato.

Rejeski believes it’s too early to dismiss nanotechnology, especially when there is a research effort devoted to greening the manufacturing process. “People are going to get smarter,” he says. “No company wants to use lots of energy and lots of toxic chemicals to make nanomaterials. But developing environmentally benign processes could take ten or twenty years and much more investment.” In fact, about the same time the FOE report was released, researchers based at the Massachusetts Institute of Technology published a new method for producing carbon nanotubes in the laboratory that they say cuts energy requirements in half and reduces harmful by-products by 90% or more.^7^ However, the FOE report notes that even if tenfold decreases in energy use are eventually achieved, carbon nanomaterials will still be much more energy-intensive to produce than aluminum or steel.^1^

Bhavik Bakshi of The Ohio State University in Columbus and TERI University in New Delhi, several of whose life-cycle analyses of carbon nanofibers are cited in the FOE report, believes governments and the nanotechnology industry must quickly and significantly increase investments in greening up both manufacturing and products to avoid repeating mistakes made with earlier innovations, like asbestos and the insecticide DDT. Historically, enthusiasm for the immediate benefits of new technologies has overshadowed consideration of potential problems until they appear years later, says Bakshi, adding, “The bar needs to be set a lot higher when it comes to adopting nanoproducts.”
